# Can Global DNA Methylation Be Influenced by Polymorphisms in Genes Involved in Epigenetic Mechanisms? A Review

**DOI:** 10.3390/genes15121504

**Published:** 2024-11-24

**Authors:** Naila Francis Paulo de Oliveira, Darlene Camati Persuhn, Maria Cristina Leme Godoy dos Santos

**Affiliations:** 1 Department of Molecular Biology, Center for Exact and Natural Sciences, Federal University of Paraíba—UFPB, João Pessoa 58051-900, PB, Brazil; darlenecp@hotmail.com; 2 Department of Celular Biology, Federal University of Paraná—UFPR, Curitiba 80060-000, PR, Brazil; mariacristina@ufpr.br

**Keywords:** global methylation, epigenetic, genetic, polymorphism, DNA methylation, DNMT, MTHFR

## Abstract

Background: Global methylation refers to the total methylation in the DNA and can also be inferred from the Line 1 and Alu regions, as these repeats are very abundant in the genome. The main function of DNA methylation is to control gene expression and is associated with both normal and pathological mechanisms. DNA methylation depends on enzymes that generate the methyl radical (e.g., methylenetetrahydrofolate reductase—MTHFR) and attach this radical to the DNA (DNA methyltransferases—DNMT). Genetic variants such as single nucleotide polymorphisms (SNP) in these genes can lead to changes in the activity or expression of MTHFR and DNMT proteins and consequently influence the DNA methylation profile. This review focuses on studies investigating inter-individual variations in the global DNA methylation profile associated with genetic polymorphisms in the *MTHFR* and *DNMT* genes. Methods: A narrative review was conducted, taking into account articles published in the last 15 years. Results: It was found that the SNPs rs1801131, rs1801133 and rs1537514 in the *MTHFR* gene, rs2241531, rs2228611, rs2228612, rs21124724 and the haplotype rs2288349, rs2228611, rs2228612, rs16999593 in the *DNMT1* gene, rs2424909, rs998382, rs6058891, rs6058897, rs4911256, rs2889703 and rs1883729 in the *DNMT3B* were associated with the level of global DNA methylation, including LINE and Alu regions in different contexts. No association was found with polymorphisms in the *DNMT3A* gene. Conclusions: It is concluded that polymorphisms in the *MTHFR* and *DNMT* genes may influence the global DNA methylation profile in health, inflammation, tumours and mental illness.

## 1. Introduction

DNA methylation is the best-studied epigenetic trait and is associated with both normal and pathological mechanisms, such as embryonic development, ageing and a variety of inflammatory, tumour and mental diseases [1,2]. It refers to the presence of the methyl radical (CH_3_) in DNA and mainly occurs in position 5 of the cytosine base preceding the guanine CpG dinucleotides. This methylated nitrogen-containing base, called 5-methylcytosine (5-mC), is so common in the genome of eukaryotes that it has been labelled “the fifth base of DNA” [2]. Non-CpG methylation, i.e., cytosine residues adjacent to adenine (CpA), thymine (CpT) or other cytosine nucleotides (CpC) are less common [3].

Global methylation refers to the total methylation in the DNA. Methylation in the LINE-1 (Long Interspersed Nuclear Element-1) and Alu (highly repetitive short DNA) regions has often been used as a surrogate measure for global methylation, as these repeats are very abundant in the genome. Specific methylation refers to analyses performed at specific sites, such as the promoter regions of genes [4,5].

DNA methylation can completely inhibit or reduce gene expression by two mechanisms: 1- by forming a physical barrier that prevents transcription factors from binding to DNA, or 2- by binding methyl-binding proteins, which in turn prevent binding to transcription factors. Other functions include the suppression of retrotransposons and the inactivation of the X chromosome in women. DNA methylation is dependent on methyl radical donors and enzymes that incorporate the methyl radical into DNA [2].

Global DNA methylation can be used as an initial screen to detect pathological changes and has been associated with a variety of contexts. Global hypomethylation is a feature commonly seen in cancer and ageing. In addition, hypomethylation of the LINE-1 and Alu regions is associated with diabetes mellitus, cardiovascular disease, osteoporosis and glaucoma [6]. Techniques for the detection of global methylation can be found in detailed publications [7,8].

There is ample evidence of inter-individual variation in global and gene-specific DNA methylation associated with age, gender, ethnicity, diet, psychological stress, smoking, alcohol, physical activity, gut microbiota, infections, environmental pollution, etc. [9,10,11,12,13,14]. Studies have also shown that genetic polymorphisms may be associated with this inter-individual variation, particularly in genes coding for enzymes involved in epigenetic mechanisms, such as the generation of the methyl radical and the attachment of this radical to DNA [15]. The functional integrity of the enzymes that regulate these pathways is important to ensure adequate efficiency of these mechanisms. Variants in genes encoding regulatory enzymes are characterised by changes in the activity or expression of these enzymes that affect DNA methylation.

Based on the above, the aim of the present study was to provide an overview of the literature on polymorphisms in the genes encoding enzymes involved in the pathway that generates the methyl radical donor and methylates DNA and their association with the global DNA methylation profile. The articles included in this narrative review cover studies from the last 15 years.

## 2. Methyl Radical Generating Pathway

**Figure 1:**
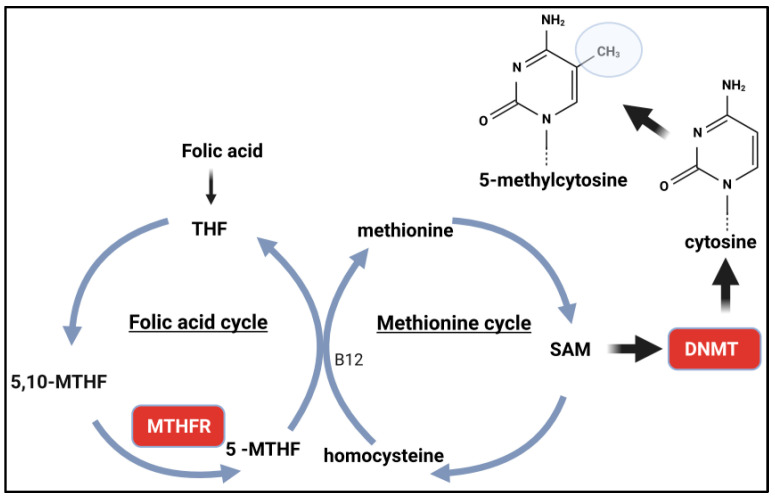
Scheme showing the formation of the methyl radical donor (SAM) up to DNA methylation. The formation of SAM depends on the folic acid cycle and the methionine cycle. The MTHFR enzyme forms 5-MTHF, which depends on vitamin B12 to regenerate methionine. Methionine is, in turn, converted into SAM. DNMT binds the CH_3_ radical of SAM to cytosines and converts them into 5-methylcytosines (5-mC). THF: tetrahydrofolate, 5,10-MTHF: 5,10-methylenetetrahydrofolate, 5-MTFH: 5-methylenetetrahydrofolate, SAM: S-adenosylmethionine. MTHFR: methylenetetrahydrofolate reductase enzyme, DNMT: DNA methyltransferase enzyme.

Methyl radical (CH_3_) donors are S-adenosylmethionine (SAM) radicals produced by the folic acid cycle. Folic acid is converted into 5,10-methylenetetrahydrofolate (5,10-MTHF) and is involved in two different metabolic processes in the cytosol: (1) DNA synthesis in thymidylate production, where 5,10-MTHF acts as a methyl donor for the conversion of deoxyuridine 5′-monophosphate (dUMP) to deoxythymidine 5′-monophosphate (dTMP; thymidylate), and (2) methylation reactions that support the methionine cycle in which methylenetetrahydrofolate reductase (MTHFR) reduces 5,10-MTHF to 5-MTHF. The 5-MTHF serves as a methyl donor to regenerate methionine from homocysteine (HCY) in a reaction catalysed by methionine synthase (MS) in a cobalamin (vitamin B12)-dependent reaction. The methionine is converted into S-adenosylmethionine (SAM). SAM is the most important methyl donor and is used by methyltransferases (MT) specific for DNA, RNA and proteins (Figure 1). During the methylation reactions, SAM is demethylated to S-adenosylhomocysteine (SAH), which is then hydrolysed to HCY [16,17].

Methylenetetrahydrofolate reductase is a flavoprotein that plays a central role in the folate cycle. It catalyses the conversion of 5,10-MTHF to 5-MTHF. The homodimeric protein contains a bound prosthetic FAD group on each domain and uses NADPH as a reducing agent. The binding of SAM allosterically inhibits the activity of the enzyme by binding to the regulatory domain [18]. Considering the important contribution to the availability of the biologically active form of folate, genetic variants of *MTHFR* have been studied for decades. Furthermore, considering that methylation processes depend on the formation of SAM, which in turn indirectly depends on the availability of 5-methylfolate, it would be reasonable that any effect on the activity or expression of MTHFR would reveal an impact on global DNA methylation.

### Polymorphisms in the MTHFR Gene and Global DNA Methylation

The polymorphisms in the *MTHFR* gene that were analysed with regard to their association with the global methylation profile are listed in Table 1. These polymorphisms are single nucleotide polymorphisms (SNP) found in the promoter, exons, introns and 3′-UTR (Untranslated Region) regions, and some of them have a previously unknown function.

Table 2 shows the extraction of data from the articles, which are listed in order of publication [19,20,21,22,23,24,25,26,27,28,29,30,31,32,33,34,35,36,37,38,39,40,41,42,43,44,45,46,47,48,49,50,51]. The most analysed polymorphism is rs1801133 (C677T). This single nucleotide polymorphism causes the replacement of Ala222Val, is related to the T allele and leads to the formation of a thermolabile molecule with a loss of ~70% of catalytic activity [52].

When analysing exposure to benzene for its carcinogenic potential, it has been shown that carriers of the rs1801133 TT genotype tend to have lower levels of global methylation and greater induction of micronuclei compared to carriers of the rs1801133 C allele [41]. This finding may be related to folate metabolism, which, when impaired, reduces the conversion of uracil to thymine, leading to misincorporation of uracil into DNA and consequently favouring chromosomal breaks [53], which could lead to an important mechanism favouring cancer [17].

As expected, an association between the homozygous rs1801133 TT genotype and lower levels of global methylation was found in studies with different designs, such as in samples from Russian blood donors [33]. Other studies have demonstrated the association of rs1801133 T with the methylation level of LINE-1 in samples from different clinical conditions. In patients with schizophrenia, no association between LINE-1 methylation and the rs1801133TT genotype was observed in males, while lower global methylation was found in females and the rs1801133TT genotype, even when folate levels were controlled [27]. LINE-1 methylation levels were also significantly lower in patients carrying the T allele with or without coronary atherosclerosis [37]. When analysing patients diagnosed with hypertension with and without medication, the TT genotype was only associated with lower methylation levels in patients without medication [48]. Another study looking at medication use showed that lower levels of global methylation were associated with the rs1801133 TT genotype in patients with recurrent glioblastoma who were or were not treated with intranasal perillyl alcohol [44]. Common to these studies is that the effect of the SNP appeared to be associated with variables such as gender [27] or medication [44,48]. The data from studies with medication suggest that the ability of a drug to alter the methylation profile needs to be better explored. Indeed, many drugs have been tested for this purpose, but little is known about the drugs that are already widely used [54].

Following this line of reasoning, two studies analysing peripheral blood found lower levels of global methylation in samples from mothers of Croatian Down syndrome carriers in the LINE-1 sequences [35] and from Brazilians in the ALU sequences [49]. While this result in the Croatian study was associated with a lower estimated folic acid consumption and the rs1801133 T allele [35], the Brazilian study showed a higher folic acid level and a lower level of global methylation without influence of the rs1801133 genotype on the result. Here, the comparison is between two studies with the same clinical condition but differing in experimental design. While Mendes et al. [49] collected data from postpartum women at the first postnatal consultation, Božović et al. [35] do not clarify the timing of recruitment. While the Croatian study estimates folic acid intake based on the consumption of certain foods, Mendes et al. use the blood dose of this metabolite. The different analytical approaches may help to explain the discrepant results on the modifying effect of folic acid levels and genotypes. However, it is also necessary to take into account the complexity of the metabolic pathway involved, the necessary and unquantified cofactors and the different genetic backgrounds. Regarding the complex pathway, a study of pregnant women in the first trimester showed an association of the T allele with lower peripheral blood methylation levels only in mothers with vitamin B6 deficiency [22].

It seems at least partially plausible to hypothesise that the rs1801133 genotype influences MTHFR activity but that clinical, environmental, nutritional, genetic and other unforeseen factors may also influence this relationship. One way to establish this relationship would be through intervention studies, which are currently scarce in the literature. An experimental approach in humans has shown that high-dose folic acid (5 mg/dL) has global effects on the sperm methylome of infertile men [34]. Reduced representation bisulfite sequencing (RRBS), which encompassed the methylation status of ∼1.8 million CpGs, revealed a significant loss of methylation in intergenic regions, introns and exons, and the observed response was quite different among *MTHFR* genotypes. Global methylation loss in sperm of *MTHFR* 677 CC males was significant only in intergenic regions, while the presence of at least one T allele (CT or TT) was associated with significant methylation loss in all sequenced tiles in promoters, exons, introns and intergenic regions. According to the authors, possible regulatory effects could occur that impair the MTHFR activity of carriers of the TT genotype and reduce the availability of methyl groups from folic acid, which would impair the cellular methylation capacity. In a zebrafish *mthfr* model (mimicking the rs1801133CT or TT genotype), the effect of folic acid supplementation, in addition to reducing the SAM:SAH ratio, showed a relevant increase in cystathionine concentration that was not observed in the *mthfr* control, which could explain the effects [55].

The lack of correlation between the rs1801133 TT genotype and DNA methylation levels was also observed in other experimental designs. A subsample of the ‘Healthy Survey of São Paulo study, representative of the population of São Paulo/Brazil, analysing food consumption, plasma markers of one-carbon metabolism and global methylation and polymorphisms of interest, revealed an age-dependent variation in methylation levels, with younger individuals showing lower levels than adults and the elderly. However, no correlation was found when rs1801133 genotype groups were compared [47]. In samples from healthy vegetarian Indian individuals, no association was also found between rs1801133 genotypes and methylation levels regardless of age group and vitamin B12 status [50]. Other studies conducted with blood or oral mucosal samples from healthy populations, including studies of adolescents, adults, men and women with their newborns, also failed to detect associations of global and LINE-1 methylation with rs1801133 [21,23,24,28,29]. In clinical tumour diseases such as colorectal cancer, breast cancer and acute lymphoblastic leukaemia, there is also no correlation between genotype and methylation [20,26,43,51]. In the study of children with acute lymphoblastic leukaemia, the outcome of oral mucositis was associated with different levels of methylation, suggesting hypomethylation in the mucosal recovery phase, although the *MTHFR* gene polymorphism had no effect [51]. In studies looking at colorectal cancer and breast cancer, there was no association between tumour and global and LINE-1 methylation in peripheral blood [20,26,43]. It is possible that studies using samples taken directly from the tumour may show some correlation, as changes in the DNA methylation profile are one of the hallmarks of cancer [56]. In other diseases, such as autoimmune diseases, cerebrovascular diseases and dementia, no associations between global methylation and rs1801133 genotype or clinical condition and global methylation have been observed either [25,31,39]. In a study of patients with diabetes, no association was found between methylation and genotype. However, higher methylation levels were observed in untreated diabetic patients than in treated patients, suggesting that treatment may influence this profile [38].

Surprisingly, there are also reports of a correlation between variants with lower MTHFR activity and increased global methylation. In healthy women, systemic inflammation, indicated by higher serum C-reactive protein, is weakly associated with global DNA hypermethylation of leukocytes in peripheral blood. This association was more evident in individuals carrying the minor allele of *MTHFR* rs1801133 [40]. Another study in which samples from pregnant women with pre-eclampsia were analysed in comparison to healthy pregnant women showed that cases carrying the CT genotype rs1801133 had significantly higher global DNA methylation than CT-matched controls. According to the authors, this suggests that women carrying heterozygotes with hyperglobal DNA methylation are more susceptible to pre-eclampsia [42]. It is worth noting that in the study conducted with pregnant women, the rs1801133 genotype was not analysed, as there were no controls in this genotype group [42]. Along the same lines, but outside the inflammatory context, are results with samples from various studies conducted in the UK, which also indicate a link between the rs1801133 TT genotype and higher levels of global methylation. In this study, intervention with riboflavin was able to reduce the previously elevated homocysteine levels in this test group but had no effect on LINE-1 hypermethylation [46]. In relation to these results, it is important to emphasise that in the studies by Nojima et al. [40] and Mishra et al. [42], a chronic inflammatory state is present, as pre-eclampsia is strongly associated with an increase in inflammatory markers, including C-reactive protein [57]. In the study by Amenyah et al. [46], the description of the population is not clear enough in terms of inclusion criteria, but the rs1801133 TT group has a higher percentage of hypertensives and higher homocysteine levels, a biomarker associated with markers of inflammatory factors including C-reactive protein [58]. This profile was also found in tumour samples and adjacent non-neoplastic skin from kidney transplant patients [19]. Kidney transplantation involves a number of pathophysiological changes from donor to recipient, and studies have discussed the presence of subclinical inflammation in these patients [59]. Given this background, it can be speculated that the global hypermethylation effect appears to be associated with a pattern that favours inflammation. Furthermore, this finding is independent of the method used to analyse global methylation, as it occurred in studies where the method was ELISA [42], luminometric [40] or pyrosequencing of LINE regions [19,46].

The rs1801131 also affects MTHFR activity but to a lesser extent than the rs1801133. rs1801131 causes the Glu429Ala (A1298C) mutation associated with the C allele and has a less significant effect on enzymatic activity [60].

In breast tissue samples from healthy women, carriers of the rs1801131 C allele showed 4% lower LINE-1 methylation [36]. Samples from a group of patients with non-syndromic orofacial clefts showed lower levels of global methylation in oral mucosa samples than the control group. However, rs1801131 CC was associated with lower LINE-1 methylation levels in the control group but not in the cases [45]. On the other hand, the rs1801131 C allele was associated with a higher percentage of methylation in DNA samples extracted from leucocytes of prehypertensive and hypercholesterolaemic individuals than the group carrying the AA genotype [30]. Similarly, the AC genotype is associated with higher methylation levels in the LINE-1 regions in the peripheral blood of patients with colorectal cancer with advanced clinical and pathological stages and aged over 61 years [43]. It is noteworthy that the two studies presenting results in which the rs1801131 CC genotype is associated with lower global methylation levels did not analyse samples of leukocyte DNA but of breast [36] and oral cavity [45]. None of these studies observed the effects of the rs1801133 genotype on global methylation levels.

As with rs1801133, no correlation was found between the rs1801131 polymorphism and DNA methylation levels in other experimental designs. In a case-control study comparing blood samples from patients with and without a breast cancer diagnosis, the rs1801131 genotype had no effect on the level of LINE-1 methylation, which in turn was lower in the clinical samples of the case group [32]. Other studies using samples of peripheral blood or oral mucosa from patients with colorectal cancer, breast cancer, acute lymphoblastic leukaemia, autoimmune diseases, cerebrovascular diseases, benzene-exposed workers, mothers of children with Down syndrome and healthy individuals also found no association with global, LINE-1 or Alu methylation [20,22,24,25,26,31,40,41,47,49,51].

Two studies analysed further SNPs in addition to rs180133 and rs180131. Wernimont et al. [21] investigated the association of methylation in the LINE-1 and Alu regions with the SNPs rs12121543, rs13306556, rs1537516, rs17367629, rs17421462, rs1994798, rs3737965, rs4846049 and rs6541003 in the peripheral blood of healthy men and found no association. Deroo et al. [32] analysed the SNPs rs3737967 and rs1537514 in the peripheral blood of healthy women with sisters who had breast cancer, and rs1537514 was associated with increased LINE-1 methylation and a lower risk of breast cancer. However, the authors do not clarify which allele or genotype is associated with this profile. This SNP is located in the 3′-untranslated region, and predictions in SNPinfo indicate that it is a putative microRNA binding site, suggesting that these polymorphisms are associated with changes in the expression of MTHFR.

In summary, an intuitive conclusion based on the functionality of SNPs in *MTHFR* suggests that reduced enzyme activity (or reduced expression) could lead to reduced SAM levels and, consequently, reduced DNA methylation. Indeed, some studies show this, but others show a correlation with higher methylation levels. The data from these studies show that other factors may contribute to this profile or predominate over genotype. Other studies have also shown that the intuitive association depends on a reduction in the levels of certain substrates that act in the folic acid cycle.

## 3. Enzymes That Insert the Methyl Radical into DNA

Enzymes from the DNA methyltransferase (DNMT) family use the SAM radical to methylate DNA. In humans, this family of enzymes consists of DNMT1, DNMT3A, DNMT3B and DNMT3L. It has also been shown that DNMT3A is expressed in the form of two splice isoforms: DNMT3A1 and DNMT3A2, the latter being shorter by 219 N-terminal amino acid residues [61]. DNMT3L have no catalytic function but plays a role in the establishment of DNA methylation through recruitment or activation of *de novo* DNMTs [62]. While DNMT1 acts on newly synthesised hemimethylated DNA strands after DNA replication to maintain the methylation profile in daughter cells, DNMT3A and B add CH_3_ to regions without prior methylation marks and are therefore known as *de novo* methylases [63]. Nevertheless, some studies have shown that DNMT3A and B can also ensure the maintenance of a current DNA methylation level [64,65].

**Figure 2:**
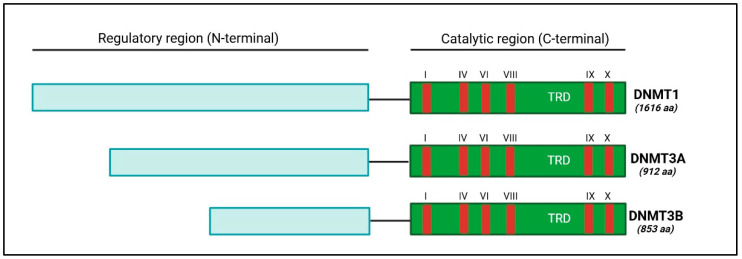
Structural domains of the three isoforms of DNMT with catalytic function. N-terminal: Amino-terminal, C-terminal: Carboxy-terminal, TRD: Target Recognition Domain, responsible for DNA recognition, aa: amino acid. The C-terminal region presents six conserved motifs (I, IV, VI, VIII, IX and X). The N-terminal region shows great variability between the different DNMTs and has the ability to distinguish between partially methylated and non-methylated DNA strands.

The structure of DNMT enzymes comprises a regulatory (N-terminal) and a catalytic (C-terminal) region, the latter having 10 small motifs, six of which are highly conserved among DNA methyltransferases, especially in DNMT1, DNMT3A and B (motifs I, IV, VI, VIII, IX and X) (Figure 2) [66].

DNMTs scan the DNA, recognise CpG sites and introduce 2’-deoxycytidine into the catalytic pocket of the enzyme. A cysteine residue binds to position 6 of the cytosine; then, the methyl group is transferred from the SAM to the C5 position of the cytosine, resulting in 5-methylcytosine (5-mC) (Figure 1). The cysteine is then released by β-elimination, and the enzyme is ready to start a new catalytic cycle [67].

The importance of DNA methyltransferases for mammalian development is emphasised by the fact that genetic deletion of one of these enzymes in mice leads to pronounced developmental defects, including lethal phenotypes. In humans, embryonic stem cells lacking DNMT1 are not viable [63].

Since the integrity of DMNT activity is extremely important for the maintenance of DNA methylation, it is reasonable to assume that changes in the activity or expression of these proteins may influence the DNA methylation profile.

### Polymorphisms in the DNMT Genes and Global DNA Methylation

The polymorphisms in the *DNMT* genes that were analysed with regard to their association with the global methylation profile are listed in Table 3. These polymorphisms are single nucleotide polymorphisms (SNP) found in the promoter, exons, introns and 3′-UTR (Untranslated Region) regions, and some of them have a previously unknown function.

Table 4 shows the data extraction from the articles listed in order of publication [20,21,25,28,30,32,49,51,68,69,70,71,72]. Some of these articles also analysed SNPs in *MTHFR* and have already been discussed in this review.

In contrast to the studies on *MTHFR*, where most involve two polymorphic sites, the literature on SNPs in *DNTM* genes includes some studies with a range of polymorphisms, but few data involve the same polymorphic site. This weakens the evaluation of the data and shows that further studies on the influence of SNPs in *DNMT* and global methylation are urgently needed.

Fourteen SNPs were analysed in relation to *DNMT1* (Table 3). The *DNMT1* rs2228612 GG genotype correlated with lower levels of global DNA methylation in Japanese with or without autoimmune thyroid disease [25]. This SNP is a non-synonymous substitution (Ile—A allele—to Val—G allele) with a minor allele frequency of 5% in Japan. It is located in the region coding for the nuclear localisation sequence of the enzyme, which plays an important role in binding to CpG sites [73]. This substitution reduces the enzyme’s DNA-binding ability; therefore, DNA methylation levels may be lower in individuals with the GG genotype. The authors also point out that this genotype is associated with the persistence of autoimmune Graves’ disease, suggesting that a lower global DNA methylation capacity in individuals with this genotype could reduce the methylation levels of the *IL1B* and *TGFB* promoter regions and increase the production of these enzymes, a mechanism that should hinder the remission of Graves’ disease in patients with the *DNMT1* rs2228612 GG genotype [25].

The *DNMT1* rs2228611 GG genotype correlated with higher levels of global DNA methylation in children with haematological tumours treated with methotrexate with or without oral mucositis [51]. The bioinformatics tool SNPinfo indicates that this SNP is located in the exonic splicing enhancer region. The G allele can lead to alternative splicing and the development of different transcriptional variants of *DNMT1* [74]. Guimarães et al. (2024) [51] hypothesise that the change from G to A may alter the serine/arginine-rich protein binding activity and pre-RNA splicing of *DNMT1*, leading to a change in *DNMT1* expression. Thus, the GG genotype may be associated with increased expression of *DNMT1* and a resulting increase in global methylation. It is important to note that this SNP in the haplotype, but not individually, was associated with global DNA methylation levels in blood cells from Chinese [71]. The GGGT haplotype of *DNMT1* (consisting of rs2288349, rs2228611, rs8111085 (fused to rs2228612) and rs16999593) increases global DNA methylation, but before correction for false discovery rate. Furthermore, the same SNP was not associated with DNA methylation in Alu or LINE-1 elements in the peripheral blood of healthy men [21].

The *DNMT1* rs2114724 T allele has been associated with higher LINE-1 methylation in males but not in females [69]. It is hypothesised that the T allele confers an intronic enhancer effect that enhances *DNMT1* gene expression, which could lead to increased LINE-1 methylation [75]. A striking observation in the literature is the consistently lower LINE-1 methylation in women compared to men. Although this sex-specific difference is not entirely clear, it appears that the LINE elements are involved in the inactivation of the X chromosome, with the inactive chromosome showing hypomethylation compared to the active X chromosome. However, methylation at specific LINE-1 elements on autosomes was not methylated differently between the sexes [76].

A possible association between *DNMT1* rs2241531 G alleles of the mother and lower global methylation levels in her offspring was demonstrated. However, this result was not robust enough to be corrected for multiple tests [68]. Furthermore, Ionue-Choi et al. found no association between the same SNP and LINE-1 methylation status in healthy Chinese adults [69].

The other SNP studies showed no association with global methylation (rs11880388, rs2162560, rs2290684, rs2336691, rs7253062, rs759920, rs8101626, rs8111085 (fused to rs2228612) [20,21,30,68,71] or LINE-1 methylation (rs7253062, rs2288350) [69].

Twenty-one SNPs were examined with respect to *DNMT3A* (Table 3), but no association was found with the Alu element (rs11678631, rs11695471, rs1550117, rs6546045, rs6733868, rs7578575) [21], LINE-1 methylation (rs1550117, rs6722613, rs7575625, rs7581217, rs7587636, rs13036246, rs34048824, rs17745484) [32,69] or global methylation (rs1550117, rs7581217, rs11683424, rs13002567, rs2289195, rs2304429, rs36012910, rs734693, rs7590760) [25,30,51,72]. The *DNMT3A* rs1550117 was analysed by five authors in different populations: Brazilian, Chinese, Spanish and American, with concordant results (Table 4).

Twenty-four SNPs in *DNMT3B* were analysed (Table 3). The *DNMT3B* rs2424909 GG genotype has been associated with global hypomethylation in exposed and unexposed benzene workers [72]. This SNP creates a binding site for the glucocorticoid receptor transcription factor and deletes two binding sites for ADR1 and AP-2alph. The glucocorticoid receptor is a ligand-activated transcription factor. It not only activates enhancers containing glucocorticoid response elements (GREs) but also inhibits the action of other transcription factors, including AP1 and nuclear factor-κB (NF-κB). Changes in these transcription factor binding sites can lead to changes in *DNMT3B* expression and, consequently, the global methylation profile. Hypomethylation and rs2424909 could, therefore, regulate gene expression by altering transcription factors and influencing benzene-induced genotoxicity and carcinogenicity [72].

The *DNMT3B* SNPs rs998382, rs6058891, rs4911256 and rs2889703 were associated with global DNA methylation. Individuals with T/T, T/T, A/A and C/C, respectively, had higher global methylation levels and were associated with suicide attempts in psychiatric patients, suggesting that patients with these susceptibility genotypes have hypermethylation of their genome [70]. These *loci* include sites where splicing is regulated, binding sites for transcription factors and other regulatory regions, and could, therefore, potentially influence the expression of the DNMT3B gene and its DNA methylation activity. It is important to note that the SNPs rs998382, rs6058891 and rs4911256 are in high linkage disequilibrium with each other; therefore, one or more of these SNPs could be responsible for the association with the global methylation levels. Crescenti and co-workers [30] also observed an association between the *DNMT3B* SNP rs998382 and hypermethylation in homozygous T males consuming cocoa. However, these data should be evaluated with caution as this association did not reach statistical significance in either study after rigorous corrections for multiple tests. In addition, Wernimont and co-workers [21] could not find an association between rs6058891 and methylation in the Alu element in the peripheral blood of healthy men.

*DNMT3B* rs6058897 A maternal allele was associated with lower global methylation levels in their offspring, but only before correction for multiple testing [68]. The SNPs in *DNMT3B*, rs1569686, rs24242932, rs2424913, rs2424928, rs2424932, rs406193, rs437302, rs4911263, rs6058870, rs6087990, rs992472 were not associated with global methylation in one or more studies [25,28,30,49,51,68,70,72]. Similarly, SNPs rs2424914, rs2424922, rs6058869 and rs6058896 were not associated with methylation in the Alu element [21], and SNPs rs2424908, rs6141813 and rs742630 were not associated with LINE-1 methylation [32,69].

In summary, the literature showed that lower global methylation levels were associated with the *DNMT1* rs2228612 GG genotype [25] and the *DNMT3B* rs2424909 GG genotype [72] in blood cells. The maternal G and A alleles of the *DNMT1* rs22415316 and *DNMT3B* rs6058897 SNPs, respectively, were associated with lower levels of global methylation in the umbilical cord of their offspring [68]. For the Alu region, the *DNMT3B* SNP rs1883729 AA genotype and lower vitamin B6 were associated with lower levels of global methylation in blood cells [21]. On the other hand, higher global methylation levels were associated with the *DNMT1* rs2228611 GG genotype in oral mucosal cells and the rs21147724 T allele in the LINE-1 region in blood cells [51,69]. In addition, higher global methylation has been associated with the *DNMT1* GGGT haplotype (rs2288349, rs228611, rs811085 and rs16999593) [71], *DNMT3B* rs998382 TT, rs6058891 TT, rs4911256 AA and rs2889703 CC genotypes [30,70], but only before corrections by multiple testing.

Several other SNPs in *DNMT1* and *DNMT3B* were not associated with global methylation (see Table 4). None of the studies showed an association between global methylation and SNPs in *DNMT3A*. The observations from the literature provide only limited evidence that *DNMT* SNPs influence interindividual variation in global DNA methylation, and further studies are urgently needed.

## 4. Final Considerations

**Figure 3:**
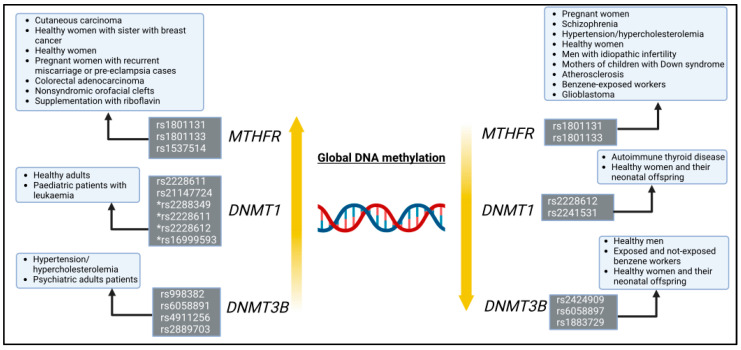
Summary of results regarding the increase or decrease in global DNA methylation levels and polymorphisms in the *MTHFR* and *DNMT* genes in health conditions, inflammatory, tumour and mental illness. * haplotype-dependent variation.

The present study found that polymorphisms in genes encoding enzymes involved in epigenetic mechanisms, such as the generation and attachment of the methyl radical to DNA, can influence the global DNA methylation profile. The polymorphisms rs1801131, rs1801133 and rs1537514 in the *MTHFR* gene, rs2241531, rs2228611, rs2228612, rs21124724 and the haplotype rs2288349, rs2228611, rs2228612, rs16999593 in the *DNMT1* gene, rs2424909, rs998382, rs6058891, rs6058897, rs4911256, rs2889703 and rs1883729 in the *DNMT3B* gene were associated with the level of global DNA methylation, including the LINE-1 or Alu regions. It is clear that this correlation depends on the context since the same polymorphism studied in different contexts does not have the same correlation. For example, the *MTHFR* polymorphisms rs1801131 and rs1801133 and *DNMT1* rs2228612 are associated with both higher and lower global methylation levels (Figure 3). Another factor that could explain the differences is the techniques used to analyse global methylation. Different techniques have a more or less sensitivity and specificity for the detection and measurement of global DNA methylation. In ascending order of accuracy, ELISA, pyrosequencing, HPLC, and mass spectrometry can be mentioned [7,8,77]. Another point is that different techniques measure methylation in different ways, e.g., LUMA measures CpG methylation, while HPLC or mass spectrometry measure all cytosine methylation, including non-CpG methylation [77]. Non-CpG methylation is less common and can be found in cells with high *de novo* methylation activity, such as oocytes, embryonic stem cells and brain tissue [3]. None of these cell types were investigated in the studies discussed in this review. Furthermore, even if global methylation can be inferred from the LINE and Alu regions (comprising about 45% of the human genome) [78], it is not exactly a total DNA methylation, which could also explain the differences in the results. Overall, all these aspects must be taken into account to compare the results between the studies.

Some factors such as age, levels of inflammatory proteins, cigarette and alcohol consumption, folic acid levels and others may contribute as confounding factors, and in some cases, the association was not confirmed after adjusting for these factors. Interestingly, no association was found with polymorphisms in the *DNMT3A* gene. Another point to consider is that DNA methylation is tissue-specific and often reflects the pathological state of the tissue, so the genotype may not have an influence in this situation. Therefore, studies with pathological conditions as well as healthy populations are important to understand the relationship between genetics and epigenetics, as the data presented in this review show that associations occur in health, inflammation, tumours and mental conditions. Another observation is that most studies on *MTHFR* investigated two polymorphic sites, whereas for *DNMT,* the same polymorphic site was rarely investigated in more than one study.

Since global DNA methylation levels do not reflect site-specific methylation, it is possible that the SNPs discussed here are associated with changes in the site-specific methylation profile, e.g., in promoter regions, and consequently lead to changes in gene expression. Indeed, some studies show an association between polymorphisms and gene-specific methylation, a topic that also requires careful consideration. Furthermore, since SAM is a methyl radical donor for enzymes that methylate histones (histone methyltransferases -HMTs) [16], it is possible that polymorphisms in genes involved in the generation of methyl radicals also affect histone methylation, another epigenetic mechanism that controls gene expression and is already associated with disease. It is important to note that the removal of the methyl radical from the DNA is performed by the ten-eleven translocation (TET) enzyme family [2]. If polymorphisms in *DNMT* genes can influence the DNA methylation profile, it stands to reason that this also applies to polymorphisms in *TET*. However, no study has yet addressed this question.

The data presented here show that polymorphisms in the *MTHFR* and *DNMT* genes can influence the global methylation profile and are valuable candidates for further investigation in health, inflammation, tumours and mental illness.

## Figures and Tables

**Table 1:** A description of the polymorphisms in genes that encode MTHFR identified in the studies.

Gene/Polymorphism	Variation	Polymorphism Location	Aminoacid Change	Predicted Functionality
***MTHFR***				
rs1801131	1298 A>C	exon	Glu429Ala	C allele with associated with reduced enzyme activity
rs1801133	677 C>T	exon	Ala222Val	T allele with associated with reduced enzyme activity
rs12121543	1167−76 G>T	intron	--	Unknown
rs13306556	1632+225 G>A	intron	--	Unknown
rs1537516	G>A	3′-UTR	--	Putative microRNA-binding site and may be associated with changes in the expression of MTHFR
rs17367629	C>T	intron	--	Unknown
rs17421462	G>/>T	intron	--	Unknown
rs1994798	1166+31 C>T	intron	--	Unknown
rs3737965	−336 C>T	promoter	--	May affect transcriptional activity
rs4846049	2572 G>T	3′-UTR	--	T allele is a micro-RNA binding site which inhibits MTHFR expression
rs6541003	G>/>C	intron	--	Unknown
rs3737967	5488 C>T	3′-UTR		Unknown
rs1537514	4869 C>G	3′-UTR	--	Putative micro-RNA-binding site and may be associated with changes in the expression of MTHFR

*MTHFR*: methylenetetrahydrofolate reductase (chr1:11785723-11805964). --: no aminoacid change. These SNPs were studied in references [19,20,21,22,23,24,25,26,27,28,29,30,31,32,33,34,35,36,37,38,39,40,41,42,43,44,45,46,47,48,49,50,51]. For details, please see Table 2.

**Table 2:** Characteristics of the studies addressing global DNA methylation and polymorphisms in the *MTHFR* gene.

Country, Year [Reference]	Study Design	Sample	Gene/Polymorphism	Tissue	Methylation/ Technique	Conclusion
Ireland, 2010 [19]	Adults with cutaneous squamous cell carcinoma in renal transplant patients	33	*MTHFR*: rs180133	Tumour and adjacent non-neoplastic skin	LINE-1/ Bisulfite pyrosequencing	MTHFR rs1801133 T allele was associated with higher levels of LINE-1 methylation in tumour and adjacent non-neoplastic skin
USA, 2010 [20]	Adults with colorectal cancer	172	*MTHFR*: rs180133, rs180131	Peripheral blood	LINE-1/ Bisulfite pyrosequencing	no association
USA, 2011 [21]	Healthy men	628	*MTHFR*: rs12121543, rs13306556, rs1537516, rs17367629, rs17421462, rs1801133, rs1994798, rs3737965, rs4846049, rs6541003, rs1801131	Peripheral blood	LINE-1 and Alu/ Bisulfite pyrosequencing	no association
Mexico, 2012 [22]	Pregnant women	195	*MTHFR*: rs1801133, rs1801131	Peripheral blood	Global/ LUMA	MTHFR rs1801133 T allele was associated with lower global methylation levels in women with vitamin B6 deficiency
Brazil, 2012 [23]	Healthy adults	159	*MTHFR*: rs1801133	Peripheral blood	Global/ ELISA	no association
United Kingdom, 2012 [24]	Healthy women and their neonatal offspring	201 mother-child pairs	*MTHFR*: rs1801133, rs1801131	Peripheral blood (mothers) cord blood (neonates)	Global/ LUMA	no association
Japan, 2012 [25]	Adults with autoimmune thyroid disease	53	*MTHFR*: rs180133, rs180131	Peripheral blood	Global/ ELISA	no association
Japan, 2012 [26]	Women with breast cancer	383	*MTHFR*: rs1801133, rs1801131	Peripheral blood	Global/ ELISA	no association
USA, 2012 [27]	Adults with schizophrenia with antipsychotic treatment for at least the previous 6 months	133	*MTHFR*: rs1801133	Peripheral blood	LINE-1/ Bisulfite pyrosequencing	MTHFR rs1801133 TT was associated with lower global methylation in women
Spain, 2013 [28]	Healthy adults	892	*MTHFR*: polymorphism not mencioned	Peripheral blood	LINE-1/ Pyrosequencing	no association
Brazil, 2013 [29]	Healthy adults	54	*MTHFR*: rs1801133	Oral mucosa	Global/ ELISA	no association
Spain, 2013 [30]	Pre-hypertensive, stage-1 hypertensive or hypercholesterolemic adults who consumed cocoa	214	*MTHFR*: rs180133, rs180131	Peripheral blood	Global/ HPLC	Trend of the MTHFR rs1801131 AA genotype to be associated with lower levels of global methylation in men
Germany, 2014 [31]	Family-based study with index cases of cerebrovascular disease	313	*MTHFR*: rs1801133, rs1801131	Peripheral blood	Global/ Cytosine-extension assay	no association
Porto Rico, 2014 [32]	Women with full or half-sister with breast cancer but had not been diagnosed with the disease themselves.	940	*MTHFR*: rs1801133, rs1801131, rs3737967, rs1537514	Peripheral blood	LINE-1/ Pyrosequencing	MTHFR rs1537514 was associated with increased LINE-1 methylation and reduced risk of breast cancer
Russia, 2014 [33]	Healthy women	160	*MTHFR*: rs1801133	Peripheral blood	Global/ ELISA	MTHFR rs1801133 TT genotype was associated with lower global methylation levels
Canada, 2015 [34]	Healthy normozoospermic men presenting idiopathic infertility with 6 months of high-dose folic acid supplementation	30	*MTHFR*: rs1801133	Sperm	LINE-1 and DMRs of imprinted loci/ Bisulfite pyrosequencing	MTHFR rs1801133 T allele was associated with lower global methylation levels across promoters, exons, introns and intergenic region in sperm after high-dose folic acid supplementation in healthy men
Croacia, 2015 [35]	Mothers of children with Down syndrome and mothers of children without Down syndrome or other aneuploidy	194	*MTHFR*: rs1801133	Peripheral blood	LINE-1/ qPCR	MTHFR rs1801133 T allele and low dietary folate was associated with lower LINE-1 methylation levels in both mothers
USA, 2015 [36]	Healthy women	121	*MTHFR*: rs1801133, rs1801131	Breast tissues	LINE-1/ Bisulfite pyrosequencing	MTHFR rs1801131 C allele was associated with lower LINE-1 methylation levels
China, 2016 [37]	Adults with coronary atherosclerosis	210	*MTHFR*: rs1801133	Peripheral blood	LINE-1/ Pyrosequencing	MTHFR rs1801133 T allele was associated with lower LINE-1 methylation levels in patients with or without coronary atherosclerosis
Africa, 2016 [38]	Diabetes status in mixed ancestry subjects	564	*MTHFR*: rs1801133	Peripheral blood	Global/ ELISA	no association
Poland, 2016 [39]	Adults with dementia	194	*MTHFR*: rs1801133	Peripheral blood	Global/ ELISA	no association
Japan, 2018 [40]	Healthy women	384	*MTHFR*: rs1801133, rs1801131	Peripheral blood	Global/ LUMA	MTHFR rs1801133 T allele was associated with higher levels of global methylation in healthy women with higher levels of C-reactive protein
China, 2018 [41]	Benzene-exposed workers	512	*MTHFR*: rs1801133, rs1801131	Peripheral blood	Global/ ELISA	Trend of the MTHFR rs rs1801133 TT genotype to be associated with lower levels of global methylation in workers exposed to benzene
India, 2019 [42]	Pregnant women with recurrent miscarriage cases and pre-eclampsia cases	133	*MTHFR*: rs1801133	Peripheral blood	Global/ ELISA	MTHFR rs1801133 CT genotype was associated with higher levels of global methylation in women with pre-eclampsia cases
Brazil, 2019 [43]	Adults with colorectal adenocarcinoma	102	*MTHFR*: rs1801133, rs1801131	Tumor samples	Global/ ELISA	MTHFR rs1801131 AC genotype was associated with higher levels of global methylation in individuals aged over 61 years in clinicopathological staging III and IV
Brazil, 2020 [44]	Adults with recurrent glioblastoma under perillyl alcohol intranasal treatment	100	*MTHFR*: rs1801133	Peripheral blood	Global/ ELISA	MTHFR rs1801133 TT genotype was associated with lower levels of global methylation in patient with or without intranasal treatment
Chile, 2020 [45]	Paediatric patients with nonsyndromic orofacial clefts	190	*MTHFR*: rs1801133, rs1801131	Oral mucosa	LINE-1/ Bisulfite pyrosequencing	MTHFR rs1801131 AC genotype was associated with higher levels of LINE-1 methylation in controls but not in cases.
Ireland, 2020 [46]	Adults supplemented with riboflavin	160	*MTHFR*: rs1801133	Peripheral blood	LINE-1/ Bisulfite pyrosequencing	MTHFR rs1801133 TT genotype was associated with higher levels of LINE-1 methylation; Riboflavin supplementation reduced these levels.
Brazil, 2020 [47]	Healthy children and adults exposed to mandatory flour fortification with folic acid	442	*MTHFR*: rs1801133, rs1801131	Peripheral blood	Global/ HPLC	no association
India, 2021 [48]	Hypertensive patients with or without treatment	481	*MTHFR*: rs1801133	Peripheral blood	Global/ ELISA	MTHFR rs1801133 TT genotype was associated with lower global methylation level in hypertensive patients not on medication
Brazil, 2021 [49]	Mothers of children with Down syndrome and mothers who had at least one child without Down syndrome or any other aneuploidy	167	*MTHFR*: rs1801133, rs1801131	Peripheral blood	LINE-1 and Alu/ Bisulfite pyrosequencing	no association
India, 2024 [50]	Healthy adults	1095	*MTHFR*: rs1801133	Peripheral blood	Global/ ELISA	no association
Brazil, 2024 [51]	Oncopediatric patients with oral mucositis	76	*MTHFR*: rs1801133, rs1801131	Oral mucosa	Global/ ELISA	no association

LUMA: Luminometric Methylation Assay; ELISA: Enzyme-Linked Immunosorbent Assay; HPLC: High Performance Liquid Chromatography; qPCR: quantitative polymerase chain reaction; DMRs: Differentially methylated regions.

**Table 3:** A description of the polymorphisms in genes that encode DNMTs identified in the studies.

Gene/Polymorphism	Variation	Polymorphism Location	Aminoacid Change	Predicted Functionality
***DNMT1***				
rs2290684	3394+34T>C	intron	--	Disrupts/creates splicing regulatory element
rs2241531	1043+26 G>C	intron	--	No functional evidence
rs2114724	1832+14 T>C	intron	--	T allele is suspected to confer an intronic enhancer effect, enhancing gene expression
rs2288350	684-82 G>A	intron	--	Unknown
rs7253062	G/A	intron	--	Unknown
rs2228611	32204 A/G	exon	Pro453Pro	G allele can lead to alternative splicing and to the development of several transcription variants of DNMT1
rs2228612/rs8111085	9794 A>G	exon	Ile327Val	Reduces the ability to bind to CpG regions
rs2162560	A>G	intron	--	A allele is suspected to confer an intronic enhancer effect, enhancing gene expression
rs759920	649-197 T>C	intron	--	Effect of the variant on RNA or protein function
rs16999593	290 A>G	exon	His97Arg	May affect the structure and function of DNMT1
rs11880388	G>A	intron	--	Unknown
rs8101626	G>A	intron	--	Predicted to affect the transcriptional regulation of DNMT1 mRNA
rs2288349	2721-45 C>T	intron	--	No functional evidence
rs2336691	A/G	intron	--	Potential regulatory on the stability of DNA molecule, or regulate gene transcription and expression by generating intronic miRNA
***DNMT3A***				
rs1550117	−2448 A>G	promoter	--	A allele may be associated with increased expression of DNMT3A
rs6722613	G>A	intron	--	Unknown
rs7575625	A>G	intron	--	Unknown
rs7581217	C>T	intron	--	Unknown
rs7587636	G>A	intron	--	Unknown
rs13036246	C>T	intron	--	Unknown
rs34048824	T>C	intron	--	Unknown
rs7590760	G>C	intron	--	G allele may be associated with increased expression of DNMT3A mRNA levels
rs36012910	−2720 A>G	promoter	--	May affect expression of DNMT3A
rs147001633	2645 G>A	exon	Arg882His	R882H mutation (A Alelle) led to an 80% reduction DNMT3A in activity
rs2304429	2597+30 C>T	intron	--	CC genotype may be associated with increased expression of DNMT3A
rs2289195	2173+26 C>T	intron	--	Intronic enhancer
rs13002567	T>C	intron	--	Unknown
rs734693	2083-272 C>T	intron	--	Unknown
rs11683424	C>T	intron	--	May be correlated with the alternative splicing
rs11678631	A>G/A>T	intron	--	Unknown
rs11695471	T>A/T>C	intron	--	Unknown
rs6546045	C>T	intron	--	Unknown
rs6733868	C>G/C>T	intron	--	Unknown
rs7578575	T>A/T>C	intron	--	Unknown
rs17745484	C>T	intron	--	Unknown
***DNMT3B***				
rs992472	C>A	intron	--	Unknown
rs2424928	1906-5T>C	intron	--	Unknown
rs2424932	827A>G	3’-UTR		Creates glucocorticoid receptor and heat shock transcription factor binding site
rs6058897	A/C	not reported		Unknown
rs437302	G>/>C	not reported		Unknown
rs406193	T>A/T>C/T>G	not reported		Unknown
rs1569686	−579 G>T	promoter		May affect promoter activity
rs2424913	−149 C>T	promoter	--	T allele may be associated with increased expression of DNMT3B
rs2424908	C>G/C>T	intron	--	Unknown
rs6141813	A>G	intron	--	Unknown
rs998382	A>G	intron	--	May act as an internal promoter of DNMT3B
rs2889703	A>C	intron	--	Disrupts splicing regulatory element, promoter, transcription factor binding site
rs6058891	C>T	exon		Disrupts/creates exonic splicing
rs4911256	A>G	intron	--	Unknown
rs6087990	−283 T> C	promoter	--	T allele may be associated with reduced expression of DNMT3B
rs2424909	−579 G>T	promoter		Creates/disrupts transcription factors binding sites
rs4911263	2302-212 T>C	intron	--	Unknown
rs1883729	G>/>C/G>T	intron	--	Unknown
rs2424914	A>C/A>G/A>T	intron	--	Unknown
rs2424922	1674 T>C	exon	Tyr558Tyr	Creates an exonic splicing enhancer site
rs6058869	C>T	promoter	--	
rs6058896	C>T	3’-UTR	--	Disrupts a micro-RNA site, disrupts/creates transcription factors binding sites
rs6058870	G>/>T	promoter	--	Unknown
rs742630	C>A/C>G	intron	--	Unknown

*DNMT1*: DNA methyltransferase 1 (chr19:10133346-10194953); *DNMT3A*: DNA methyltransferase 3A (chr2:25227874-25341925); *DNMT3B*: DNA methyltransferase 3B (chr20:32762385-32809356); --: no aminoacid change. These SNPs were studied in references [20,21,25,28,30,32,49,51,68,69,70,71,72]. For details, please see Table 4.

**Table 4:** Characteristics of the studies addressing global DNA methylation and polymorphisms in genes encoding DNMTs.

Country, Year [Reference]	Study Design	Sample	Gene/Polymorphism	Tissue	Methylation/ Technique	Conclusion
USA, 2020 [20]	Adults with colorectal cancer	172	*DNMT1*: rs8111085 (merged into rs2228612)	Peripheral blood	LINE-1/ Bisulfite pyrosequencing	no association
USA, 2011 [21]	Healthy men	628	*DNMT1*: rs8111085 (merged into rs2228612), rs11880388, rs2162560, rs2228611, rs8101626 *DNMT3A*: rs11678631, rs11695471, rs1550117, rs6546045, rs6733868, rs7578575 *DNMT3B*: rs1883729, rs2424914, rs2424922, rs6058869, rs6058891, rs6058896	Peripheral blood	LINE-1 and Alu/ Bisulfite pyrosequencing	*DNMT3B* rs1883729 AA genotype and lower vitamin B6, was associated with lower methylation in Alu element
Japan, 2012 [25]	Adults with autoimmune thyroid disease	53	*DNMT1*: rs2228612, rs16999593 *DNMT3B*: rs1569686	Peripheral blood	Global/ ELISA	*DNMT1* rs2228612 GG genotype was associated with lower global methylation level in individual with or without autoimmune thyroid disease
Spain, 2013 [28]	Healthy adults	892	*DNMT3A*: rs11683424, rs7581217, rs1550117	Peripheral blood	LINE-1/ Pyrosequencing	no association
Spain, 2013 [30]	Pre-hypertensive, stage-1 hypertensive or hypercholesterolemic adults who consumed cocoa	214	*DNMT1*: rs2162560, rs759920, rs7253062 *DNMT3A*: rs2304429, rs2289195, rs13002567, rs734693 *DNMT3B*: rs998382, rs4911263, rs2424932	Peripheral blood	Global/ HPLC	Trend of the *DNMT3B* rs998382 TT genotype to be associated with higher levels of global methylation in men who consumed cocoa, only before multiple testing corrections.
England, 2013 [68]	Healthy women and their neonatal offspring	137 mother-child pairs	*DNMT1*: rs2290684, rs2241531 *DNMT3B*: rs992472, rs2424928, rs2424932, rs6058897, rs437302, rs406193 rs1569686, rs2424913	Peripheral blood (mothers) cord blood (neonates)	Global/ LUMA	Trend of the *DNMT1* rs2241531 G and *DNMT3B* rs6058897 A maternal alleles to be associated with lower global methylation level in their offspring, only before multiple testing corrections.
China, 2013 [69]	Healthy Adults	440	*DNMT1*: rs2114724, rs2241531, rs2288350, rs7253062 *DNMT3A*: rs1550117, rs6722613, rs7575625, rs7581217, rs7587636, rs13036246, rs34048824 *DNMT3B*: rs2424908, rs6141813	Peripheral blood	LINE-1/ Bisulfite pyrosequencing	*DNMT1* rs2114724 T allele was associated with higher LINE-1 methylation in men
Ireland, 2013 [70]	Psychiatric adults patients attempter and non-attempter to suicide	144	*DNMT3B*: rs24242932, rs998382, rs2889703, rs6058891, rs4911256,	Peripheral blood	Global/ ELISA	Trend of the *DNMT3B* rs998382 TT, *DNMT3B* rs6058891 TT, *DNMT3B* rs4911256 AA and *DNMT3B* rs2889703 CC genotypes to be associated with higher global methylation in psychiatric adults patients, only before multiple testing corrections.
Porto Rico, 2014 [32]	Women with full or half-sister with breast cancer but had not been diagnosed with the disease themselves.	940	*DNMT3A* rs13036246, rs7575625, rs17745484 *DNMT3B*: rs742630	Peripheral blood	LINE-1/ Pyrosequencing	no association
China, 2016 [71]	Healthy adults	309	*DNMT1*: rs2288349, rs2228611, rs8111085, rs16999593, rs2336691	Peripheral blood	Global LC-ESI-MS/MS	*DNMT1* rs2288349, rs2228611, rs8111085, and rs16999593 GGGT haplotype was associated with higher global methylation level in healthy adults, only before multiple testing corrections
China, 2017 [72]	Benzene-exposed workers	512	*DNMT3A*: rs36012910, rs1550117, rs147001633 (also known as R882H) *DNMT3B*: rs1569686, rs2424909, rs2424913	Peripheral blood	Global/ ELISA	*DNMT3B* rs2424909 GG genotype were associated with lower global methylation level in exposed and not-exposed benzene workers
Brazil, 2021 [49]	Mothers of children with Down syndrome and mothers who had at least one child without Down syndrome or any other aneuploidy	167	*DNMT3B*: rs1569686, rs2424913, rs6058870	Peripheral blood	LINE-1 and Alu/ Bisulfite pyrosequencing	no association
Brazil, 2024 [51]	Oncopaediatric patients with oral mucositis	76	*DNMT1*: rs2228611 *DNMT3A*: rs7590760, rs1550117 *DNMT3B*: rs6087990, rs2424913	Oral mucosa	Global/ ELISA	*DNMT1* rs2228611 GG genotype was associated with higher global methylation level in pediatric oncology children with or without oral mucositis.

ELISA: Enzyme-Linked Immunosorbent Assay; LUMA: Luminometric methylation assay; LC-ESI-MS/MS liquid chromatography-electrospray ionisation/tandem mass spectrometry; HPLC: High Performance Liquid Chromatography.

## Data Availability

All the data are contained in the manuscript file and will be made available from the corresponding author upon reasonable request.
